# Biotechnological, Nutritional, and Therapeutic Applications of Quinoa (*Chenopodium quinoa* Willd.) and Its By-Products: A Review of the Past Five-Year Findings

**DOI:** 10.3390/nu16060840

**Published:** 2024-03-14

**Authors:** Rhaira Fernanda Ayoub Casalvara, Bruna Mayara Roldão Ferreira, José Eduardo Gonçalves, Natália Ueda Yamaguchi, Adelar Bracht, Lívia Bracht, Jurandir Fernando Comar, Anacharis Babeto de Sá-Nakanishi, Cristina Giatti Marques de Souza, Rafael Castoldi, Rúbia Carvalho Gomes Corrêa, Rosane Marina Peralta

**Affiliations:** 1 Programa de Pós-Graduação em Tecnologias Limpas, Universidade Cesumar—UNICESUMAR, Maringá 87050-900, Brazil; rhaira.casalvara@gmail.com (R.F.A.C.); jose.goncalves@unicesumar.edu.br (J.E.G.); natalia.yamaguchi@unicesumar.edu.br (N.U.Y.); rubia.correa@unicesumar.edu.br (R.C.G.C.); 2 Programa de Pós-Graduação em Ciência de Alimentos, Universidade Estadual de Maringá, Maringá 87020-900, Brazil; pg55114@uem.br (B.M.R.F.); jfcomar@uem.br (J.F.C.); absnakanishi@uem.br (A.B.d.S.-N.); 3 ICETI—Instituto Cesumar de Ciência, Tecnologia e Inovação, Maringá 87050-390, Brazil; 4 Programa de Pós-Graduação em Bioquímica, Universidade Estadual de Maringá, Maringá 87020-900, Brazil; abracht@uem.br (A.B.); lbracht@uem.br (L.B.); cgmsouza@uem.br (C.G.M.d.S.); rcastoldi@uem.br (R.C.)

**Keywords:** quinoa, bioactive compounds, sustainability, agro-industrial wastes, upcycling

## Abstract

This study aimed to provide an updated critical review of the nutritional, therapeutic, biotechnological, and environmental aspects involved in the exploitation of *Chenopodium quinoa* Willd and its biowastes. Special attention was devoted to investigations of the therapeutic and nutritional properties of different parts and varieties of quinoa as well as of the use of the biowaste resulting from the processing of grain. Studies published from 2018 onward were prioritized. Extracts and fractions obtained from several *Chenopodium quinoa* matrices showed antioxidant, antidiabetic, immunoregulatory, neuroprotective, and antimicrobial effects in in vitro and in vivo models and some clinical studies. The activities were attributed to the presence of phytochemicals such as polyphenols, saponins, peptides, polysaccharides, and dietary fibers. Quinoa wastes are abundant and low-cost sources of bioactive molecules for the development of new drugs, natural antioxidants, preservatives, dyes, emulsifiers, and carriers for food and cosmetics applications. Among the demands to be fulfilled in the coming years are the following: (1) isolation of new bioactive phytochemicals from quinoa varieties that are still underexploited; (2) optimization of green approaches to the sustainable recovery of compounds of industrial interest from quinoa by-products; and (3) well-conducted clinical trials to attest safety and efficacy of extracts and compounds.

## 1. Introduction

In recent decades, the diet of the Western world has been enriched by the arrival of some neglected and underutilized plant species (NUS). Prior to this, NUS were almost exclusively produced and consumed in their places of origin. These cultures are currently attracting great interest, especially from health-conscious consumers interested in their bioactive components. Due to the growing popularity and associated marketing, several NUS are now given the status of “superfoods”, which further fuels demand and, thus, increases prices. This rapid and important increase in production is what we call the “boom” of a crop [1,2,3,4].

Various NUS cereals are suitable for growing in climates or conditions deemed “inappropriate”, and for that reason, they must be explored, developed, and valued now more than ever. Due to their resilience to the most diverse biotic and abiotic stress scenarios, underutilized cereals may be the answer to the alarming state of world food security, while meeting the nutritional demands of a growing population. Such objectives reflect the current global sustainability agenda in food and nutrition security [1,5].

The species *Chenopodiumm quinoa* Willd., popularly called quinoa, is an example of NUS that has experienced its “boom” in recent years. This dicotyledonous annual plant, considered the “mother grain” by the South Americans (Incas), has an agricultural history that goes back more than 5000 years in the Andes region (Bolivia, Peru, Ecuador). Because of its advantages of cold hardiness, drought resistance, and easy cultivation, quinoa has been widely planted around the world as a “super crop” [6,7]. The dry seed is consumed as a cereal grain and stands out for its excellent nutritional value. The balance of essential amino acids, fatty acids, micronutrients, vitamins, and antioxidants is considered to be of high quality compared with the main cereals. As a gluten-free food grain with a low glycemic index, this pseudocereal is an alternative in special diets and industries [8,9]. Quinoa seeds are considered healthy ingredients and their extracts and isolated compounds have antidepressant, antiviral, anticancer, and anti-inflammatory properties [5,10].

The world production of quinoa soared from 32,435 tons in 1961 to 161,415 tons in 2019, and the harvested area has increased substantially, from 52,555 hectares in 1961 to 184,585 hectares in 2019 [11]. Although more than 70% of the global exports are still supplied by Peru and Bolivia, the production outside the Andes area is increasing every year. Currently, *C. quinoa* is cultivated in more than 123 countries [8,12,13].

The impressive increase in consumer awareness and, consequently, in the consumption of quinoa and derivative products, is the consequence of the extensive research efforts on this food crop, as revealed by the number of annual publications on the subject, which has practically doubled since 2018. The total number of scientific articles published in the last five years containing the term “quinoa” in the title is suggestive, surpassing 1160 articles. Of these, 48 correspond to review articles, with the majority in the Food Science and Technology search domain (obtained from the Web of Science platform, December 2022). So far, very few review papers have focused on the bias of sustainability and valorization of quinoa residues.

Considering the above, this study aimed to provide an updated critical review of the nutritional, therapeutic, biotechnological, and environmental aspects involved in the exploitation of quinoa and its biowastes. The search was performed in ScienceDirect and Web of Science databases using the keywords “quinoa” and “residue” and their variations in several combinations. Inclusion criteria were as follows: (a) investigations of the therapeutic, nutritional, or biotechnological properties of different parts of the *C. quinoa* plant; (b) proposals for valuing quinoa biowaste within the upcycling concept; (c) studies carried out during the last five years (2018 to December 2022); (d) high-quality research.

## 2. Botanic and Agronomic Features

*Chenopodium quinoa* Willd. is a native grain-like culture of the Andean region of South America that comprises Peru, Bolivia, Ecuador, Colombia, and Chile. From 3000 to 4000 years ago, quinoa was domesticated for human consumption and livestock feed. The annual herbaceous species is reputable as a pseudo-cereal since it pertains to the family of spinach and sugar beets [14,15]. *C. quinoa* shows vast genetic diversity following its adaptations to the most extreme environmental conditions inherent in its ecotypes in the Andes. Plants measure between 0.3 and 3 m and can occur in diverse colors (from white, yellow, and pink to darker red, purple, and black), with thick, erect, woody stalk and taproot systems. In early growth stages its polymorphic leaves are commonly green and turn to yellow, red, or purple with maturity. Likewise, the seed (diameter between 0.28 and 2.1 cm) holding two flat surfaces and rounded sides might be black, red, pink, orange, yellow, or white [14,16,17].

Global warming constraints the world food demand by influencing the conditions under which crops can be yielded, especially by the increase in soil salinity, which is forecasted to touch more than 50% of all arable lands by 2050 [16,18]. Thus, adaptation of agriculture to soil salinity is an area of huge scientific attentiveness, as salt stress may strongly affect plant productivity worldwide and threaten food security [19]. *C. quinoa,* whose genome was sequenced in 2017, has been recognized as a promising alternative culture for salt-affected regions: it was designated by the Food and Agricultural Organization of the United Nations (FAO) as one of the crops for biosaline agriculture that will have a key role in guaranteeing food security for future generations [17,20,21].

Halophytic plants endure salinity levels that would easily kill most conventional crops. Moreover, halophytes can remove the excess salt from soil and water while producing food. In the course of their evolution, they have developed different physiological mechanisms to cope with numerous stress conditions [19,22]. Quinoa, for instance, is particularly tolerant to abiotic stress factors such as drought, frost, and salinity [23]. Growth of quinoa in salt concentrations of up to 500 mM NaCl has been reported, with salinity levels that are equivalent to those of seawater [24].

Quinoa is a low-input crop, ideally suited to organic and low-input production systems [25]. Nevertheless, quinoa can be highly responsive to irrigation and nitrogen (N) fertilization under various agroecological conditions [2,21], which suggests yield limitations under lower nitrogen supply and drought conditions. Irrigation and N-fertilization, coupled with other agronomic benefits, have resulted in quinoa being increasingly grown with high productivity and input use, with significant increments in crop yields. Satisfactory yields can be achieved by optimizing the local production environment, such as the variety × sowing date interaction. Nonoptimal sowing dates and poor soil conditions at sowing have been shown to reduce seed number, grain yield, and grain quality [21,26,27].

The crop water productivity (CWP; the amount of biomass in kg per volume of water supply in m^3^) of the crop is low and can be increased: (a) if water loss from evaporation is reduced; (b) if the negative effect of drought stresses at specific phenological phases is avoided; and (c) if unfavorable conditions during crop growth (i.e., pests and diseases) are diminished. Furthermore, *C. quinoa* can adapt to most types of soils, but different results have been obtained when assessing crop N-uptake. Some authors argue that maximal yields are reached at 80 kg N ha^−1^, but they can differ according to the soil type, being the highest in sandy-clay-loam soils. On the other hand, some authors have acknowledged that increasing N-fertilization does not determine quinoa growth or yield [28].

## 3. Nutritive Features

The introduction of pseudocereals in food products is directly linked to their high nutritional value. The processed quinoa grain, ready for preparation and subsequent consumption, is a food rich in both macronutrients (proteins, carbohydrates, and fats) and micronutrients [13,29,30,31] (Table 1 and Table 2). Quinoa seeds contain significant amounts of thiamine, folic acid, and vitamins and are rich in bioactive compounds such as saponins, phenolic compounds, phytosterols, phytoecdysteroids, betalains, and peptides [10].

When compared with other cereals common in human consumption, quinoa stands out for its higher concentrations of lysine, methionine, and cysteine, as well as higher levels of starch, fiber, and protein [30,32]. The ash content is also higher than in rice and wheat, for example, which justifies the high presence of minerals where calcium, magnesium, and potassium are in bioavailable forms [33]. Likewise, folic acid and riboflavin levels are generally higher in quinoa than in other grains, such as oats, wheat, barley, rye, rice, and corn (Table 2). The amount of riboflavin found in 100 g of quinoa seed meets 80% of the daily needs determined for children and 40% for adults [34,35].

**Table 1:** Nutritional characterization (mean values) of Chenopodium quinoa grains from different cultivars.

Cultivar	RBQ	-	Quinoa Preta	-	BRS Piabiru	Ames 13.727	-	Jessie	-
Origin	Bolivia	USA	Peru; Spain	China	Brazil	Asia	-	Europe	Ecuador
**Nutrients ^1^ (%)**									
Energy (kcal)	-	-	424.0	-	389	345.9	359	340.7	-
Protein	12.52	10.82	14.6	71.76	17.0	14.24	13.5	15.96	16.70
Fat	6.48	4.65	6.8	1.4	6.0	10.6	6.48	5.10	-
Carbohydrate	-	74.10	76.1	-	66.5	-	57.2	50.07	-
Fiber	-	-	-	-	-	-	9.53	15.33	11.10
Ash	2.43	2.08	2.7	1.66	2.1	-	2.44	3.33	1.96
Sugars	2.36	-	3.1	-	1.17	-	4.31	2.81	-
Amide	-	-	-	22.04	-	-	-	-	-
**Minerals ^2^**									
Calcium	-	43.98	-	-	-	1.7	-	87.9	0.18
Iron	-	4.80	-	-	-	0.15	-	7.9	7.8
Magnesium	-	195.07	-	-	-	2.9	-	205.5	0.16
Potassium	-	387.28	-	-	-	16	-	908.8	0.33
Sodium	-	16.18	-	-	-	2.95	-	11.7	0.02
Phosphorus	-	599.43	-	-	-	-	-	550.3	0.32
**Amino acids ^2^**									
**EAAs**									
Histidine	-	2.98	-	3.51	-	-	0.36	7.54	2.31
Isoleucine	-	3.86	-	3.2	-	-	0.49	4.46	2.87
Leucine	-	7.01	-	5.91	-	-	0.79	5.43	-
Lysine	-	6.28	-	5.45	-	-	0.78	13.55	3.81
Valine	-	4.65	-	3.01	-	-	0.61	5.66	3.81
Threonine	-	4.21	-	2.01	-	-	0.49	7.82	2.62
Methionine + Cystine	-	-	-	1.88	-	-	0.46	1.57	-
Tryptophan	-	-	-	-	-	-	-	0.48	-
**NEAAs**									
Phenylalanine + Tyrosine	-	7.30	-	4.97	-	-	1.24	6.8	3.31
Glutamic acid	-	15.66	-	13.39	-	-	1.77	14.39	13.44
Aspartic acid	-	9.06	-	7.88	-	-	1.07	11.39	7.68
Alanine	-	4.80	-	2.68	-	-	0.55	3.51	2.87
Glycine	-	5.68	-	4.02	-	-	1.13	4.22	11.00
Arginine	-	9.32	-	7.64	-	-	0.68	7.79	6.50
Proline	-	3.93	-	2.70	-	-	0.49	-	2.87
Serine	-	4.98	-	3.12	-	-	0.61	3.95	8.19
**Fatty acids ^1^ (relative %)**									
C14:0	0.14	-	-	-	0.277	0.48	-	0.19	-
C16:0	8.86	-	20	-	11.09	12.06	-	9.02	-
C16:1	0.09	-	-	-	0.088	0.30	-	0.13	-
C17:0	0.18	-	-	-	0.058	-	-	0.06	-
C18:0	0.78	-	-	-	0.786	1.62	-	0.47	-
C18:1	29.84	-	32	-	22.67	21.9	-	17.93	-
C18:2	50.16	-	31	-	56.70	54.38	-	61.40	-
C18:3n3	6.52	-	-	-	2.74	9.11	-	7.17	-
C20:1	1.35	-	-	-	1.50	-	-	0.32	-
C20:4	-	-	-	-	-	0.40	-	0.28	-
C22:1	1.16	-	-	-	1.78	-	-	1.31	-
**References**	[36]	[37]	[38]	[39]	[40]	[41]	[42]	[43]	[44]

^1^ g/100 g edible part; ^2^ mg/100 g de DM (dry matter). EAAs = essential amino acids; NEAA = nonessential amino acids. C14:0 myristic acid; C16:0 palmitic acid; C16:1 palmitoleic acid; C17:0 margaric acid; C18:0 stearic acid; C18:1 oleic acid; C18:2 linoleic acid; C18:3n3 α-linoleic acid; C20:1 eucasonoic acid; C20:4 arachidonic acid; C22:1 erucic acid.

**Table 2:** Vitamin composition of *C. quinoa* grain in comparison with the profiles of other grains present in human food.

Vitamins (100 mg g^−1^)	Quinoa ^A^	Quinoa ^A^	Quinoa ^B^	Corn ^C^	Sorghum ^D^	Rice ^E^
Ascorbic acid	-	-	-	6.8	-	1.66
α tocopherol	1.39	0.919	2.44	0.07	0.32	4.91
β tocopherol	0.063	-	0.08	-	0.012	-
γ tocopherol	0.053	2.67	4.55	-	0.092	-
δ tocopherol	-	-	0.35	-	0	-
Thiamine	0.13	-	0.36	0.155	0.79	4.30
Riboflavin	0.03	-	0.32	0.055	0.05	1.66
Niacin	12.35	-	1.52	1.77	-	6.63
**References**	[45]	[40]	[30]	[46]	[47]	[48]

^A^: *Chenopodium quinoa* Willd BRS Piabiru, dry basis; ^B^: *C. quinoa*, dry basis; ^C^: *Zea mays* convar. *saccharata* var. *rugosa,* wet basis; ^D^: *Sorghum bicolor* L., dry basis; ^E^: *Oryza sativa* L., dry basis.

*C. quinoa* grains are good sources of fat, which makes the species an oilseed crop [41]. A compilation of the lipids identified in different pseudograin cultivars (g/100 g) is presented in Table 1. The average values of total lipid content vary, incredibly, from 1.4 to 10.6%, and polyunsaturated fatty acids and essential fatty acids, such as linoleic acid and alpha-linolenic acid, stand out [36]. Likewise, the average values of total proteins vary a lot (from 10.8 to 71.7%) (Table 1). It has been proposed that such discrepancies could be associated with edaphoclimatic conditions [39]. It has been emphasized that the chemical and nutritional composition of the seed is directly impacted by the lack of adaptation to regions that are very different from those where the cultivar originated [49].

Cookies made from *C. quinoa* grain rich in linoleic acid and alpha-linoleic acid have been introduced into the diet of healthy adults aged between 50 and 70 years [50]. After four weeks, anthropometric measurements and blood tests revealed weight loss and a reduction in circulating LDL cholesterol. This confirms previous research in which significant decreases in blood glucose and LDL cholesterol were observed after treatment of healthy adults for four weeks with bread enriched with quinoa (20 g of flour) [51]. It is usually assumed that the aforementioned bioactivities depend on compounds such as polyphenols, fatty acids, amino acids, fibers, and eventually saponins [30,52].

## 4. Phytocomponents from Quinoa with Potential to Benefit Human Health

Bioactive compounds are non-nutritive plant components with health benefits that have only recently been recognized. The groups of bioactive components in pseudocereal grains include saponins, phenolic compounds, phytosterols, phytoecdysteroids, polysaccharides, betalains, and bioactive proteins and peptides. This section will address the most studied bioactive compounds from the edible part of *C. quinoa* in recent years and will review evidence of their biological activities. Table 3 shows a compilation of recent works that report the bioactive potential of grains and products derived from different varieties of C. *quinoa* grown around the world.

### 4.1. Phenolic Compounds

Phenolic compounds (PC) are secondary metabolites of plants with bioactive potential, mainly antioxidant effects [53]. In a pioneering work on the biological potential of Brazilian quinoa, the composition of the hydroethanolic extract (ethanol/water, 80:20, *v*/*v*) from the grain was investigated [40]. Quercetin and kaempferol were the major PC in the extracts for which in vitro antimicrobial and antioxidant activities were found. The extract was effective (MICs = 1.0 µg/mL) toward *Bacillus cereus* (food isolate), *Staphylococcus aureus* (ATCC 6538), *Listeria monocytogenes* (NCTC 7973), *Escherichia coli* (ATCC 25922), *Enterobacter cloacae* (human isolate), and *Salmonella typhimurium* (ATCC 13311). Moreover, it was active against six fungi (MIC values ranging from 0.5 to 1.0 µg/mL) and was superior to the fungicide ketoconazole against *Penicillium ochrochloron* (MICs = 0.5 µg/mL). The authors believe that such antimicrobial potential may be related, inter alia, to the presence of flavonoids. It is important to add that toxicity in healthy pig liver cells was not detected. Similarly, the phenolic fraction of Peruvian quinoa grains, which was obtained using hexane and ethanol as extractor solvents, was characterized by its antioxidant action in degenerative diseases and memory deficits in vivo [54].

Nearly 40 cultivars of colored quinoa from the Altiplano region of Puno, Peru, were investigated [55]. Phenolic compound fractions were obtained using acid (HCl)-methanol/water (50:50, *v*/*v*), in addition to fractions of betalains from grains extracted with a water/methanol solution (80:20, *v*/*v*). Both fractions showed promising antioxidant capacity in the DPPH assay. Soluble phenolic compound fractions were also obtained through sequential hydrolysis (alkaline and acid) of American quinoa seeds, sprouts, and flakes [56]. These fractions were submitted to in vitro gastrointestinal digestion and subsequently evaluated by ORAC and DPPH chemical assays. The reported results indicate that quinoa flavonoids (mainly quercetin and kaempferol glycosides) are bioaccessible and that their release in the gastrointestinal tract may have strong antioxidant activity against free radicals.

**Table 3:** Biological activities of quinoa grains and products derived from different quinoa cultivars reported in the last three years. An arrow (→) before the solvent name is used to indicate that the first extraction was complemented by a second partition or that a new isolation procedure was performed.

Cultivar/Origin	Part of the Plant/Extract or Isolated Compounds	Investigated	Experimental Model	Main Findings	Ref.
White quinoa, United Arab Emirates	Water-soluble extract of whole grain fermented by *Bifidobacterium* spp.	Cytotoxic, antihypertensive, antidiabetic, and antioxidant activities	In vitro enzymatic and antioxidant assays, tumor cell lines	Solid-state fermentation improved the biological activities of quinoa; fermented grains showed significant antihypertensive and antioxidant activities.	[57]
White quinoa, Spain; white, black, and red quinoas, Bolivia; white quinoa, Peru	Grain flour ultrasonically extracted using 80% methanol → 70% acetone; extracts combined at the end	Antioxidant capacity	DPPH and ABTS radical scavenging tests; ferric ion reducing assay potential (FRAP) and ferrous ion chelation capacity	Peruvian white and Bolivian black quinoa samples showed the best antioxidant activities in all assays, with good correlations with total phenolic and flavonoid contents.	[36]
Ecuador	Quinoa protein concentrate obtained from grain flour → peptides released in digestion collected by ultrafiltration	Antioxidant and chemopreventive activities	Static in vitro gastrointestinal digestion; colon cancer cell culture; ORAC assay	Peptides < 5 kDa showed the highest antioxidant activity and peptides > 5 kDa promoted the greatest anticancer effects.	[35]
28 varieties from Bolivia, Chile, Denmark, USA, and Peru	Quinoa oil extracted with n-hexane → 3 mL methanol/water solution (4:1, *v*/*v*)	Antioxidant capacity	DPPH and FRAP chemical assays	Variability in total phenolic and carotenoid contents, which is reflected in different antioxidant capacities. Conspicuous amounts of phytosterols and squalene were detected in the samples.	[58]
Spain	Grain extracts obtained via ultrasound-assisted extraction with ethanol → concentration with butanol	Pancreatic lipase and alpha-glucosidase inhibition effects	In vitro enzymatic assays, intestinal simulatedconditions	The extracts (saponins + phenolics) had inhibitory effects on pancreatic lipase and alpha-glucosidase. While intestinal conditions worsened the inhibition of lipase, it slightly catalyzed the α-amylase.	[59]
Bolivia	Methanol extracts from whole grains and industrial by-products (mixed quinoa and amaranth flakes)	Antioxidant and antiproliferative activities	ABTS, FRAP, and CUPRAC antioxidant assays; human cervical carcinoma cell lines	The evaluated material is a significant source of antioxidant compounds and has demonstrated inhibitory activity against cancer cells at different stages.	[60]
Cultivars MengLi 1 and MengLi 2, China	Aqueous extracts of sprouted and non-sprouted quinoa seed yogurts	Antioxidant capacity in vitro	ABTS, DPPH, FRAP, and ORAC antioxidant assays	Germinated quinoa yogurts had good antioxidant capacities in all tests, with high levels of flavonoids and total phenolics.	[61]
USA	Soluble phenolic compounds obtained by sequential hydrolysis (alkaline and acid) from quinoa seeds, sprouts, and flakes	Antioxidant activity	Static in vitro gastrointestinal digestion; ORAC and DPPH assay	The results indicate that quinoa flavonoids (mainly quercetin and kaempferol glycosides) are bioaccessible and that their release in the gastrointestinal tract may have strong antioxidant activity against free radicals.	[56]
38 colorful cultivars from the Altiplano region of Puno, Peru	Free phenolics extracted from grains with acid (HCl)-methanol/water (50:50, *v*/*v*); grain betalains extracted with a water/methanol solution (80:20, *v*/*v*)	Antioxidant activity	DPPH assay; Vis-NIR diffuse reflectance spectroscopy	The study demonstrated that the total antioxidant capacity of colored quinoa seeds, including non-extractable and extractable antioxidants, can be predicted by Vis-NIR spectra coupled with chemometrics.	[55]
BRS Piabiru, Brazil	Ethanol/water extract (80:20, *v*/*v*) of the grain	Antioxidant and antimicrobial activities, hepatotoxicity	Oxidative hemolysis inhibition assay (OxHLIA) and inhibition of the production of thiobarbituric acid reactive substances (TBARS) (cell-based methods); plate microdilution and INT assay; pig liver cell culture	In this pioneering study of Brazilian quinoa, four organic acids (quinic acid as the main one), and α- and γ-tocopherols as isoforms of vitamin E were detected. Quercetin and kaempferol glycosides were the principal phenolics of the extract that presented antioxidant and antimicrobial activities, without toxic effects.	[40]
Red quinoa, Peru	Phenolic fraction obtained by extraction with hexane in Soxhlet, followed by extraction of the residue with ethanol	Neuroprotective effect	Mice with scopolamine-induced memory deficits; memory test; ex vivo acetylcholinesterase (AChE) activity, CAT and SOD activities; ROS and TBARS assays	The extract prevented induced declarative memory deficit and restored antioxidant indicators and AChE activity.	[54]
Cultivar MengLi 2, China	Oleanolic acid (OA) extracted from sprouted quinoa yogurts	Antioxidant, antidiabetic, and antiangiogenic activities	DPPH and ABTS chemical assays, in vitro inhibition of dipeptidyl dipeptidase DPP-4; human umbilical endothelial cells	Quinoa OA had its concentration optimized by the germination process and showed antioxidant, antidiabetic, and antiangiogenic activities. Sprouted quinoa can be exploited to obtain OA nutraceuticals or consumed as a food/functional ingredient.	[62]
White quinoa, China	Soluble (SDF) and insoluble (IDF) dietary fiber extracted from grain	Antioxidant and hypoglycemic capabilities	DPPH, FRAP, hydroxyl radical assays; in vitro inhibition of alpha-glucosidase and alpha-amylase; glucose adsorption capacity and glucose dialysis delay index	Polyphenols bound to insoluble fiber were responsible for the superior DPPH radical scavenging activity and α-amylase inhibitory activity compared with insoluble fiber.	[63]
Red quinoa, Spain	Saponin-rich methanolic extracts by ultrasound-assisted extraction → acid hydrolysis	Pancreatic lipase inhibitory activity and hypocholesterolemic effect	In vitro enzymatic assays; in vitro gastrointestinal digestion of cholesterol	The hydrolysis of saponin-rich extracts enhanced compounds’ bioactivities and can be used to obtain multifunctional products to inhibit pancreatic lipase and cholesterol absorption at the same time.	[64]
Argentina	Hydroalcoholic extracts of germinated quinoa grain obtained under subcritical conditions	Antioxidant activity	TEAC, FRAP, and ORAC chemical tests; Rancimat accelerated oxidation test	The authors found excellentantioxidant capabilities for extracts rich in bioactives obtained from germinated quinoa, which revealed the potential for application in food, cosmetics, and pharmaceutical products.	[65]
China	Polysaccharides isolated by membrane technology from the aqueous extract of the grain	Antioxidant, antidiabetic, and immunoregulatory potential	DPPH and ABTS tests; in vitro inhibition of alpha-glucosidase and alpha-amylase; RAW 264.7 cell cultures, and instrumental analysis	Three fractions of polysaccharides, formed mainly by glucose, galactose, and arabinose, exertedimmunoregulatory activity in RAW264.7 cells and dose-dependent in vitro antioxidant and antidiabetic activities.	[66]
MengLi, China	Polysaccharide isolated from the grain by alkaline extraction and purified with column chromatography	Antioxidant capacity in vitro	DPPH, ABTS, hydroxyl radical, superoxide radical, and ferric-reducing capacity assays	The authors obtained and characterized the structure of the polysaccharide, which showed promising radical scavenging activities in all assays performed.	[67]
Altiplano, Peru	Bioactive peptides (BP) obtained from quinoa protein → hydrolysis with alcalase and trypsin	Antioxidant activity and alpha-glucosidase inhibition effect	ABTS+ radical scavenging activity; inhibitory measurement of alpha-glucosidase enzyme in vitro	BP showed the highest antioxidant activity; the best α-glucosidase inhibitor was generated by trypsin. These preparations could be used to produce nutraceuticals or food ingredients.	[68]
Red quinoa, Peru	Grain ethanolic extract	Neuroprotective effect	*Drosophila melanogaster* with rotenone-induced Parkinson’s disease (PD); behavioral tests; dopamine dosage, AChE, CAT, and SOD tyrosine activities; ROS and TBARS assays	Quinoa extract protected against rotenone toxicity, indicating potential as an adjunct in the treatment of PD. This effect may be related to the phenolics in the extract, which showed antioxidant action and the ability to modulate some enzymes.	[69]
Canada	Bioactive peptides (BP) isolated from hulled grain protein concentrate via electrodialysis with ultrafiltration membranes	Antihypertensive and antidiabetic properties	In vitro glucose uptake;animal model of spontaneously hypertensive rats (SHR)	PB were able to modulate L6 cell glucose with a potential antidiabetic effect. A decrease in systolic blood pressure was observed in the presence of fractionated peptides, with 100 mg/kg as a dose comparable to captopril (positive control)	[70]
China	Water-soluble polysaccharides (QP) obtained by a successive extraction and purification protocol developed by the authors	Antioxidant activity; antiaging effects	ABTS and DPPH chemical tests; male Kunming mice submitted to the Morris water maze test, biochemical analysis, and histopathological and immunohistochemical analysis	QP demonstrated antiaging effects in vivo, restoring the cognitive impairment of aging induced by D-galactose. Administration of 800 mg·kg^−1^ for 10 weeks decreased oxidative damage and lipid peroxidation, improved organ function, and restored lipid metabolism and SIRT1 pathway regulation.	[71]
Egypt	Whole seeds	Hypoglycemic and antidiabetic effects	Streptozotocin-induced diabetic rats	Quinoa ingestion had a hypoglycemic and improvement effect on cholesterol, HDL, and LDL levels.	[72]

### 4.2. Saponins

Saponins form a broad group of active specific metabolites in plants. They are amphipathic molecules combining two distinct moieties: a nonpolar steroid or triterpenoid aglycone and one or two polar oligosaccharides [73]. Quinoa saponins are bitter, intervening in the food’s palatability and digestibility. Furthermore, some studies have reported the potential toxicity of these compounds owing to their hemolytic activity and ability to lower surface tension [74].

Indeed, the major part of saponins in *C. quinoa* husks is triterpenoid glycosides, which are derived from beta-vanillin and encompass sugar chains and sapogenins [75]. Despite the antinutritional factor just mentioned, a robust body of evidence shows that quinoa saponins exert anticarcinogenic, hypocholesterolemic, antiviral, immunostimulatory, neuroprotective, antifungal, antiparasitic, and anti-inflammatory effects [73,76,77]. Some authors believe that the hypoglycemic effects of quinoa triterpenoid saponins result from their inhibition of alpha-glucosidase and because the compounds improve β-cell function and diminish insulin resistance.

Recently, a deep euthetic solvent(DES)-based extraction approach (based on choline chloride and glycerol) to recover saponins from Ecuadorian quinoa cultivars was developed [77]. Among the eleven sapogenins (hydrolyzed saponins) that were detected, hederagenin and phytolaccagenic acid were the major ones in all samples. As expected, the husks from bitter seeds presented the highest saponin contents. Although the hydromethanolic solution (70:30, *v*/*v*) extracted 3 times more saponins compared with DES, the latter stabilized saponins in concentrated solutions.

Pressurized hot water extraction of saponins from quinoa husks was optimized with a maximal yield of 23.06 mg/g [75]. A total of 23 compounds, mostly triterpenoid saponins and flavonoids, were detected in the extracts using LC-MS. The in vitro glucosidase activity assay revealed that the husk extracts had superior inhibitory potential toward alpha-glucosidase compared with acarbose. Furthermore, molecular docking-based interaction analyses indicated that the foremost bioactive compounds were the triterpenoid saponins.

### 4.3. Peptides

A strategy to add value to the formulation of protein-based foods is by modifying the structure of the protein, reducing the molecular size, and producing peptides with biological activity [78]. The antioxidant activity and the α-glucosidase inhibitory activity of bioactive peptides (BP) produced from the protein of Altiplano quinoa, prepared at different times of hydrolysis using alcalase and trypsin, were investigated [68]. BP hydrolyzed by alcalase showed higher antioxidant activity in vitro, but the best α-glucosidase inhibitor was generated by hydrolysis with trypsin. The authors believe that the newly prepared peptides can be used in industries as an additive or beneficial product, either as a drug (purified form) or a food ingredient (non-purified form). BP was also isolated from the protein concentrate of husked Canadian quinoa grain using electrodialysis with ultrafiltration membranes [70]. These fractions were able to modulate L6 cell glucose, exerting a potential antidiabetic effect in spontaneously hypertensive rats (SHR). Furthermore, a decrease in systolic blood pressure was observed due to the administration of 100 mg/kg fractionated BP, a dose comparable to captopril (positive control) (Table 3).

### 4.4. Polysaccharides and Dietary Fiber

Membrane separation technology was employed to isolate polysaccharides (QP) from the aqueous extract of Chinese quinoa grains [66]. Three fractions of QP, formed mainly by glucose, galactose, and arabinose, exerted dose-dependent immunoregulatory activities in RAW264.7 cells in addition to antioxidant and antidiabetic activities in vitro. Soluble and insoluble dietary fiber fractions were obtained from white quinoa grain cultivated in China [63]. The view was expressed that polyphenols bound to insoluble fiber were responsible for the superior DPPH radical scavenging activity and α-amylase inhibitory activity compared with insoluble fiber. A protocol with successive extraction and purification steps for the recovery of soluble QP from quinoa grains was also developed [71]. The quinoa polysaccharides demonstrated antiaging effects in vivo, restoring D-galactose-induced aging cognitive impairment in male mice. Administration of 800 mg⋅kg^−1^ for 10 weeks decreased oxidative damage, reduced lipid peroxidation, improved organ function, and restored lipid metabolism and SIRT1 pathway regulation in animals (Table 3).

## 5. Biotechnological Potential of Quinoa Grains and Quinoa Biowastes

At the time of harvesting and threshing quinoa, residues known as straw and stubble are generated. In producing countries, like Ecuador, these biowastes are commonly burned, which causes pollution and represents a fire risk, in addition to causing inconvenience to the population due to smoke, not to mention ash and deterioration of soil fauna and flora [79].

Quinoa husk is a by-product obtained from the husking process before using the quinoa seed on an industrial scale. Considering the global production volume of quinoa (about 146.74 thousand metric tons), large amounts of husks (between 8 and 12%) are produced during the husking step. Currently, most of this biomass is discarded due to the presence of bitter saponin compounds [80]. Aside from saponins, quinoa husk contains polyphenolic compounds and about 31% cellulose, 32% hemicellulose, and 21% lignin [81,82,83]. However, this residual biomass is usually burned or discarded without being used; therefore, the use of quinoa husk for industrial purposes and the investigation of its applicability in agriculture are of growing interest [83].

Regarding the valorization of the aforementioned agro-industrial wastes, it has been suggested that they can be recycled into novel products with superior added value [84]. The term ‘upcycling’ has been employed when technology and innovation are used to create efficient approaches for utilizing bioresources (i.e., food residues) in a more sustainable fashion: innovative by-products for food and feed formulations, new high-value-added food and feed components, or other products from wasted food [85]. This last category might include packaging materials, commodity chemicals for other industries, biofuel or bioenergy sources, and composting materials. Thus, upcycling quinoa residues means reusing these biomaterials and assigning them a different function, not foreseen beforehand [84].

**Figure 1:**
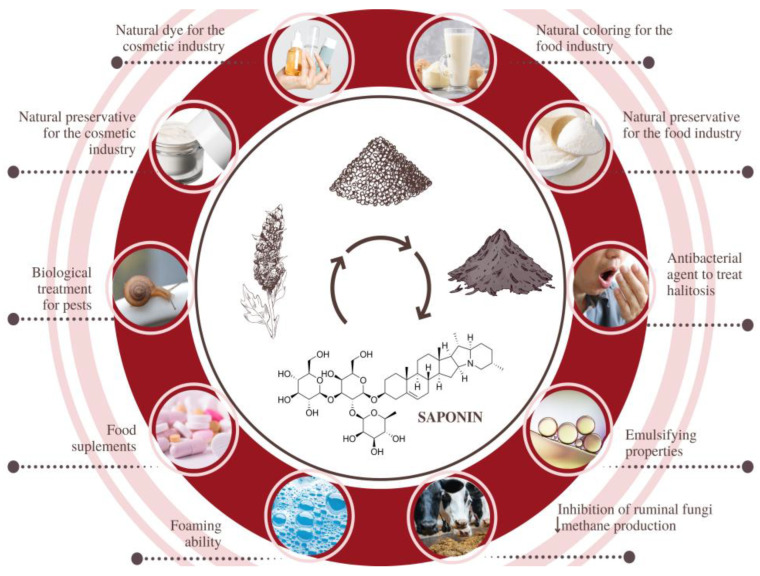
Possibilities for the upcycling of quinoa biowastes and biotechnological applications reported for grains and by-products.

Figure 1 and Table 4 bring together possibilities proposed in recent years for the upcycling of quinoa biowastes as well as several biotechnological applications reported for grains and by-products. However, other authors have reported more traditional applications, such as the use in animal nutrition supplementation [79,86] and energy production [81].

### 5.1. Food Technology

The bioactivities of dietary fibers (DF) isolated from the leaves of several Chinese quinoa cultivars were investigated [71]. Promising in vitro antioxidant activities and lipid and bile acid binding capacity were found, as well as immunoregulatory activities and prebiotic effects, which enables prospects for applications as food ingredients or nutraceuticals (in purified form). Extracts rich in betalains and saponins from the husks of colorful cultivars of Peruvian quinoa using ultrasound were prepared [90]. Characterization of these extracts revealed potential bifunctional ingredients, with coloring and preservative functions, for both food and cosmetic industries (Table 4).

A protein isolate obtained from Indian quinoa grain demonstrated excellent foaming ability, foam stability, emulsification properties, and protein solubility, indicating potential use as a natural food additive stabilizer and emulsifier [95]. Saponin-rich quinoa husk ethanolic extracts were efficient inhibitors of foodborne pathogenic bacteria, a highly desirable quality for natural preservatives [93].

### 5.2. Cosmetic and Pharmaceutical Industries

Sustainability is increasingly important to consumers, who, today, consider not only the effects that the products they buy have on their personal health but also the impact they have on the environment and on the people who work at all levels of the supply chain. As climate change has moved from a future concern to a present reality, going “green” is the driving force for innovation across all sectors, including beauty [105]. 

Surfactants are one of the main ingredients in the formulation of beauty products for forming emulsions, foams, and humectant capacity. However, the vast majority of these compounds are derived from petroleum, have a certain toxicity, and are not renewable [106,107]. Within this context, the potential of hydroethanolic extracts of Brazilian quinoa as emulsifying agents for the incorporation of vegetable oils with conditioning and fragrance properties was evaluated [92]. The surfactants showed great biotechnological potential, especially with regard to their emulsifying potential, demonstrating the feasibility of application as an additive in cosmetic products.

To simulate the possible effect of the saponin-rich extract of quinoa husk on the intercellular lipid of the *stratum corneum* (the “mortar”), the mixed lipid monolayer model, containing cholesterol, stearic acid, and ceramide VI, was used [91]. The surfactant components of the macerate, probably saponins, introduced in the subphase below the pre-compressed monolayer, significantly increased the surface pressure as well as the surface dilational modulus of elasticity. The results confirm that quinoa seed husk extracts exhibit high affinity with the cholesterol-containing intercellular lipid mixture monolayer model. This effect is probably related to the high affinity of the triterpenoid saponins, abundant in the husks, with cholesterol. This opens up countless possibilities for the use of quinoa saponins in functional cosmetic and pharmaceutical formulations.

The potential of saponins recovered from quinoa husks as efficient and affordable antibacterial agents for the treatment of halitosis was investigated [80]. It was concluded that the low polarity of the bioactives favors morphological alterations for the lysis of the membrane, which is a primary inducer of halitosis bacteria (*Fusobacterium nucleatum, Clostridium perfringens,* and *Porphyromonas gingivalis*).

### 5.3. Other Biotechnological Potentialities

*Pomacea* spp. are freshwater gastropods that significantly reduce rice productivity worldwide, especially in Asian countries. The view was expressed [87] that modified saponins from Quinoa Real boliviana husks possess a molluscicidal potential against *Pomacea maculata*, which could be safely used in water, without environmental damage. Importantly, saponins from *Chenopodium quinoa* have been approved as biopesticide ingredients by the United States Environmental Protection Agency (USEPA) [108].

The thermal properties of the inedible parts of quinoa have been studied by some authors (Table 4). In one of these studies [80], it was found that aerial parts are a better fuel than grain husks due to their calorific value (17.33 MJ/kg) and volatile matter (73.3%). Apparently, this biomass can become a new low-cost energy source for quinoa-producing countries.

Iranian quinoa husks behaved as a good substrate for bioethanol production via hydrolysis by a stable metagenome-derived laccase (isolated from a complex environment) and subsequent fermentation with *S. cerevisiae* [96]. Subsequent investigations evaluated the potential of the same biowaste as a source of cellulose for incorporation into multi-walled carbon/ZnO nanotubes [103]. It was suggested that the developed nanobiocomposite can be applied as a recyclable biocatalyst for oxidation reactions, also in high-performance fabrics and smart materials. Furthermore, the nanocellulose of the Iranian quinoa husk was an efficient carrier for the immobilization of laccase used in the removal of textile dyes [97].

## 6. Attention Points: Antinutritional Substances in Quinoa Raw Seeds and Their Use in Human and Animal Nourishment

Antinutritional factors (ANFs) are molecules that exert effects contrary to optimal nutrition, commonly reacting with nutrients and, thus, interfering with their absorption [109]. The major ANFs identified in quinoa seeds are saponins, tannins, and phytic acid, followed by nitrates, oxalates, and trypsin inhibitors. Such substances are present in higher concentrations in the outer layers of the grain (i.e., in quinoa seed husks).

There is still little information on antinutritional and/or potential toxic effects that might compromise quinoa’s nutritional quality [110]. For instance, the outcomes of prolonged saponin ingestion on human health are still not well known [111]. However, it is well established that saponins, the major ANFs in quinoa seeds, present high foaming capacity in aqueous solutions, hemolytic activity when in direct contact with blood cells, and the ability to form complexes with cholesterol and other steroidal components of the cell membranes. For these reasons, saponins are useful for crop protection against microbial infection and insect and bird herbivory activities, facilitating organic production [112]. But the use of raw quinoa seed flour in human nutrition, as well as its utilization along with seed husks in feed formulas for monogastric animals, both significant sources of surfactant and molluscicidal compounds, require some caution. As these molecules bind cholesterol within the cell membranes, they can potentially damage the gastrointestinal tract cells. Thus, the small but constant inhibition of alpha-glucosidase, together with the potential of cellular disintegration by binding to cholesterol in cells, could prompt changes preceding food intolerance, permeable intestine, and dysbiosis in humans, which are known to be irreversible.

In order to minimize the above-cited risks, thermal treatment of quinoa seeds is highly recommendable. A recent and pioneering study investigated the effects of moist heating (boiling and autoclaving) and dry heating (roasting and microwave processing) on the phenolic and flavonoid contents, antioxidant activities, ANFs (saponin and phytate contents), and functional properties of quinoa grains [113]. Thermally treated grains displayed significant reductions in both emulsification properties and flour dispersibility. Boiling (15 min) and autoclaving significantly diminished the saponin contents by 64 and 13%, respectively, whereas both dry heating methods led to increments in saponin levels [113]. The raw and treated quinoa grains can be classified as bitter once the saponin contents are within the range of 1.4–23 mg/g. Boiled grains, however, are converted into sweet quinoa [30] The observed reduction of saponins during boiling is likely due to their leaching into cooking water as quinoa saponins contain glycosides that are water soluble. Furthermore, the phytate content found in all treated and nontreated grains was much lower than the harmful range (10–60 mg/g) [113].

## 7. Final Considerations and Perspectives

**Figure 2:**
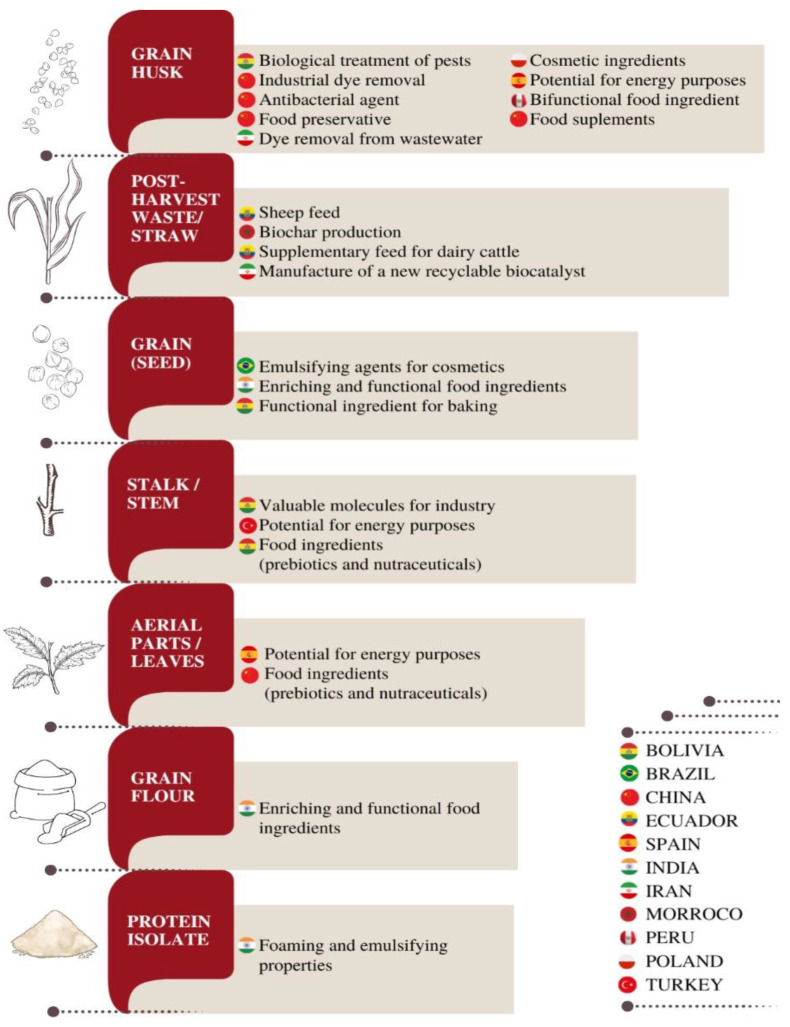
Current and potential uses of the distinct parts of the *Chenopodium quinoa* plant and its major derived products around the world.

In the last five years, research groups have reported the potential therapeutic effects of extracts obtained from different parts of dozens of varieties of *Chenopodium quinoa* cultivated around the world, and most interestingly, from its agro-industrial bioresidues. These abundant and low-cost biomasses are not only promising sources of bioactive molecules for the development of new antidiabetic, antioxidant, and antimicrobial drugs but also great matrices for obtaining natural antioxidant ingredients, preservatives, colorants, emulsifiers, and carriers for food and cosmetic applications (Figure 2). In spite of the presence of antinutritional substances in raw quinoa seeds and correspondent by-products (husks), such molecules can be easily inactivated or reduced to safe health levels via thermal processing approaches that enhance both technological and nutritional features, promoting quinoa potentiality as a functional food ingredient. Among the gaps that science must seek to fulfill in the coming years, we highlight (1) the isolation of new bioactive phytochemicals from quinoa varieties that are still underexploited; (2) the optimization of green approaches for the sustainable recovery of target compounds of industrial interest from quinoa by-products; and (3) well-conducted clinical trials to confirm the safety and efficacy of extracts and compounds isolated from these matrices.

## Figures and Tables

**Table 4:** Several biotechnological applications of quinoa grains and by-products reported in the last three years.

Cultivar/Origin	Part of the Plant/Extract	Potential Application and Justification	Ref.
Quinoa Real, Bolivia	Husk saponins, extracted with distilled water and modified by alkali treatment	Biological treatment of pests. Due to the toxicological properties and field effectiveness of the modified saponins, this molluscicide is a good candidate for the control of *Pomacea maculata*, without causing environmental damage.	[87]
China	Grain husks subjected to carbonization and chemical activation with KOH to produce porous carbon material	Removal of industrial dyes by adsorption. The biomaterial showed high adsorption rates for malachite green, rhodamine B, methyl violet, methylene blue, and methyl orange, dyes present in dyeing wastewater.	[88]
Real Blanca, Bolivia	Saponins, xylan, and cellulose from quinoa stalks	Valuable molecules for the pharmaceutical, cosmetic, and food industries. Within the biorefinery approach, integrated processing to separate saponins, xylan, and cellulose is possible.	[89]
Five colorful cultivars, Peru	Quinoa husks extracted with ultrasound for the recovery of betalains and saponins	Natural and bifunctional ingredients (coloring function + preservative function) for the food and cosmetic industries.	[90]
Ancovinto ecotype, Chile	A saponin-free extract obtained from the grain	Food preservation. An innovative glazing system based on the inclusion of hydroethanolic extracts of saponin-free quinoa promoted the quality enhancement of frozen Atlantic mackerel.	[74]
Faro variety, Poland	Aqueous husk extracts rich in saponins	Cosmetic ingredients. Saponin-rich quinoa extracts can enhance skin penetration for cosmetic or pharmaceutical purposes.	[91]
No. Jingli-1, China	Quinoa husk saponins modified by alkali treatment	Antibacterial agent to treat halitosis. Interesting due to its excellent cost-effectiveness and notorious activity against three bacteria related to halitosis.	[80]
Ecuador	Post-harvest and threshing residues submitted to the drying	Sheep feeding. The use of 20% of quinoa residue in the diet of sheep can improve the productive parameters, possibly due to an improvement in the ruminal environment.	[86]
Spain	Aerial plant parts and seed husks	Both biomaterials showed potential for energy purposes. Aerial parts were shown to be a better fuel due to their calorific value (17.33 MJ/kg) and volatile matter (73.3%).	[81]
Brazil	Grain hydroethanolic extract	Emulsifying agents for the formulation of cosmetics.	[92]
China	Quinoa husks submitted to ultrasonic extractions with 75% anhydrous ethanol	Food preservative. Due to their great cost-effectiveness and antimicrobial potential, these saponins can be used against foodborne pathogenic bacteria.	[93]
Turkey	Dried stalks/stems	Quinoa stalks could be used for energy production; the authors found an energy value of 18.27 MJ/kg for the biowaste.	[94]
Various cultivars, India	Grains, flour and protein isolates	Enriching and functional food ingredients. One of the protein isolates demonstrated excellent foaming and emulsification properties.	[95]
Iran	Quinoa rusks	Substrate for the production of bioethanol via hydrolysis with laccase (isolated from a complex environment) and subsequent fermentation with *S. cerevisiae.*	[96]
Iran	Quinoa husk nanocellulose as a carrier for laccase immobilization	Removal of dyes from wastewater. Nanocellulose was tested as a nanocarrier for laccase immobilization and provided an efficient nanobiocatalyst for the removal of malachite green and congo red from water.	[97]
Red quinoa Real, Bolivia	Fractions of polyphenols and dietary fiber extracted from grains	Functional ingredients for baking. The incorporation of 5% of dietary fiber in bread allowed for increases in total fiber content, nutritional value, and antioxidant potential.	[98]
Nine different cultivars, China	Soluble dietary fiber isolated from leaves	Functionalizing or nutraceutical food ingredients. Dietary fibers had a remarkable effect on in vitro antioxidant activities, lipid and bile acid binding capacity, immunoregulatory activities, and prebiotic effects.	[99]
Ecuador	Quinoa straw transformed into biochar (for soil correction)	Biochar was efficient in reducing cadmium bioaccumulation in leaves and provided minerals to increase productivity.	[100,101]
Morocco	Quinoa waste, dried at 40 °C for three days and then ground	Substrate for biochar production via anaerobic digestion and pyrolysis. The simultaneous use of bioproducts promoted a substantial increase in tomato production.	[102]
Ecuador	Quinoa straw and stubble (waste from the threshing process)	Supplementary feed for dairy cattle. Partial replacement of commercial feed supplementation by a combination of silage-containing quinoa straw (20%) did not affect production indicators.	[79]
Iran	Cellulose fibers recovered from quinoa waste incorporated with multi-walled carbon/ZnO nanotubes (bio-nanocomposite)	Polymer for manufacturing a new recyclable biocatalyst for oxidation reactions. High conversions and excellent selectivity were verified for the oxidation of alcohols; the biocomposite can be applied in high-performance fabrics and smart materials.	[103]
China	Saponins recovered from quinoa husks by hot pressurized water extraction	Dietary supplements to control postprandial hyperglycemia and drug potential. The in vitro inhibition of alpha-glucosidase by saponins was higher than the value recorded for acarbose; molecular docking indicated triterpenoid saponins as the most bioactive.	[75]
White Real, Bolivia	Xylooligosaccharides (XOS) from quinoa stalks	Prebiotic and nutraceutical food ingredients.	[104]
